# Rescripting Memory, Redefining the Self: A Meta-Emotional Perspective on the Hypothesized Mechanism(s) of Imagery Rescripting

**DOI:** 10.3389/fpsyg.2018.00581

**Published:** 2018-04-20

**Authors:** Alessandra Mancini, Francesco Mancini

**Affiliations:** ^1^Scuola di Specializzazione in Psicoterapia Cognitiva S.r.l., Associazione di Psicoterapia Cognitiva, Rome, Italy; ^2^Department of Technology, Communication and Society, Guglielmo Marconi University, Rome, Italy

**Keywords:** imagery rescripting, autobiographical memory, self-representation, psychopathology, emotional invalidation, secondary problem, meta-emotional problem

## Abstract

Imagery Rescripting (ImRs) is a therapeutic technique that aims to reduce the distress associated with negative memories of early aversive experiences. It consists of prompting patients to *rescript* the autobiographical memory in line with their unmet needs. In recent years, ImRs was found effective in reducing symptoms of disorders such as depression, social phobia, obsessive-compulsive disorder, post-traumatic stress disorder, and personality disorders. However, the cognitive mechanisms underlying such broad effectiveness are currently an object of debate. Empirical evidence has shown that ImRs reduces the negative self-belief derived from aversive memories in different types of mental disorders. However, existing accounts are not very accurate in explaining how this change in self-belief occurs and therefore why ImRs is effective across psychopathologies. We propose that ImRs changes the semantic self-representation encapsulated in the aversive memory by reducing the meta-emotional problem (i.e., perceiving a negative emotion as problematic and unacceptable). Empirical evidence implicates the meta-emotional problem or “secondary problem” in the maintenance of different disorders and has shown that treating it leads to symptoms reduction. Here we hypothesize that: (i) ImRs as a stand-alone treatment may lead to a reduction of symptoms; negative self-belief and the meta-emotional problem; and (ii) the reduction of the meta-emotional problem might mediate the relation between symptoms and negative self-belief reduction. To test our hypothesis, we present an experimental procedure that could be used in future studies. We conclude discussing the existing theoretical frameworks that attempt to unravel the mechanisms that play a role in ImRs.

## Introduction

Early traumatic experiences are considered to increase vulnerability to psychopathology by classical cognitive models of emotional disorders underlying cognitive behavioral therapy (CBT) (Beck, 1976; Beck et al., 1985; Foa and Kozak, 1986; Wells, 1997; Harvey et al., 2004). Childhood aversive events can be considered traumatic in two ways. In the stronger sense, the trauma corresponds to single or repeated episodes of abuse or violence. In the weaker sense, trauma corresponds to experiences of lower emotional intensity over a longer time, such as a disorganized/unsure type of attachment between parents and children. According to both perspectives, traumatic experiences interfere with the self. For some authors, traumatic experiences interfere with the self as a process by disorganizing its structure and eliciting dissociation (van der Kolk et al., 1996; Liotti, 2004). In contrast, we agree with those positions that see trauma as interfering with self-representation (i.e., a form of semantic memory that describes the quality associated with the self, Brewin, 2006). Specifically, the type of attachment to primary relational figures plays a pivotal role in forming “Internal Working Models,” “Internal Schemas,” or “Early Maladaptive Schemas” which trigger and maintain psychopathology (Beck, 1964; Lorenzini et al., 1985; Lorenzini and Sassaroli, 1995; Young et al., 2003). Accordingly, a vast body of literature indicates that issues in self-concept and emotional regulation are associated to “emotional invalidation” during childhood (Drea, 2016; Witkowski, 2017), which consists of the invalidation, negation, or trivialization of a child’s emotions and thoughts by caregivers (Linehan, 1993). These early aversive experiences are often associated with intrusive imagery and linked to distressing autobiographical memories. Imagery rescripting (ImRs) is a therapeutic technique, used in the context of Schema Therapy and CBT, that aims to reduce distress associated with these memories (Arntz and Weertman, 1999) and change their meaning (Arntz, 2011).

The classic procedure (Arntz and Weertman, 1999) consists of three phases. First, the patient is asked to enter a distressing memory with a similar emotional content of that characterizing current symptomatology. Ideally, the memory should belong to an event that occurred during childhood (see Ehlers et al., 2005 for examples of the procedure used in case of traumatic events occurred during adulthood). In this phase, the therapist encourages the patient to talk in the present tense from the child’s perspective. After the factual details are clear, the therapist asks the child about his/her emotions and needs. Then, the rescripting begins and the patient is asked to step into the image as an adult and to take care of the child-self. This may involve the prevention of abuse, the creation of a safe environment for the child, and doing whatever the adult feels is right in that situation considering the child’s needs. In the third phase, the child can ask the adult for further intervention until her/his needs are fully met.

A prominent feature of ImRs is its efficacy across psychopathologies such as: personality disorders (PD), post-traumatic stress disorder (PTSD), social anxiety disorder (SAD), body dysmorphic disorder, bulimia nervosa, depression, and obsessive-compulsive disorder, both as part of a treatment package (Arntz, 2012) and, more recently, as a stand-alone treatment (Morina et al., 2017). However, despite these promising results, the cognitive mechanisms underlying such broad effectiveness are currently an object of debate.

Several studies support the view that ImRs impacts self-representation. Specifically, ImRs reduced the strength of negative self-beliefs encapsulated in aversive memories of socially anxious participants (Wild et al., 2007, 2008; Lee and Kwon, 2013). Unfortunately, in these studies, ImRs was always preceded by cognitive restructuring, making it difficult to derive conclusions about ImRs’ direct impact. ImRs’ efficacy as a stand-alone treatment was investigated for SAD, revealing a significant reduction in participants’ ratings of the validity and accuracy of their memory-derived core beliefs, as well as in the content of these beliefs, which was revised following ImRs (Reimer and Moscovitch, 2015). Similarly, a single session of ImRs diminished negative self-belief in bulimia patients when compared to a control condition that consisted of verbally examining the effects of beliefs on current functioning, including dieting. Emotional (and rational) negative self-belief reduction was associated with mood and behavior change, including a decreased urge to binge (Cooper, 2011). Finally, a recent study assessed the impact of ImRs on self-structures as measured by state self-esteem; self-concept clarity; self-description consistency, on memory characteristics (i.e., vividness and distress) and on affective measures. At follow-up, participants rated the memory as less important for their sense of self than at the first ImRs session. They also reported higher state self-esteem and positive affect, as well as reduced negative affect and anxiety after recalling the memory (Çili et al., 2017). Taken together, these results seem to indicate that ImRs strongly affects what a person thinks or has learned about him/herself (i.e., self-representation). A change in self-representation could explain ImRs’ trans-diagnostic effectiveness, however, *how* does this change occur? Here, we propose that ImRs facilitates a change in self-belief *by* modifying the appraisal that the patient has learned to make about his/her own aversive emotions. Indeed, negative beliefs about aversive emotions may determine a secondary emotional response that might exacerbate and maintain the primary reaction and the consequent regulation attempts (Greenberg and Safran, 1990; Ellis, 1999; Greenberg, 2002; Hayes et al., 2006; Mennin and Farach, 2007; Clark and Beck, 2010). Indeed, perceiving an emotion as problematic, aversive, or unacceptable instead of normal, comprehensible, and acceptable can influence the way a person regulates the emotional state itself (Gardner et al., 1988; Hofmann, 2013). This phenomenon, which has been defined as “*secondary problem* or *meta-emotional problem*” (Ellis, 1980, 2003), has been considered by Clark and Beck (2010) as one of the most relevant factors in psychopathology. Indeed, the authors claimed that: “*the greatest differences between clinical and non-clinical anxiety are evident in the secondary, strategic controlled processes responsible for the persistence of anxiety.*” (p. 53).

## Authors’ Hypothesis

Traditional ImRs procedure implies: (i) a change in perspective from that of the child to that of the adult and (ii) the attempt to meet the child’s unmet needs (Arntz and Weertman, 1999). Depending on the situation this could result in different types of actions (e.g., nurturing; protecting, soothing, empowering, etc.), however, in order to meet the needs of the child-self, the adult-self must first adopt an empathic disposition and acknowledge the child’s-self affective state. Indeed, clinical observation suggests that if the patient does not legitimate his/her own feelings it is very unlikely he/she will complete the exercise at all. In our perspective, this attitude of the adult-self could validate the child-self’s suffering. Therefore, the new meaning that could emerge from ImRs is that child’s suffering was legitimate, adequate, and deserving of care. In our view, this would be in contrast with the meaning inferred during childhood in attachment relations (i.e., the invalidation of the child negative emotions, Gardner et al., 1988). Indeed, in a meta-emotional perspective, if an individual believes that experiencing negative emotions is problematic, those could become aversive events, which amplify emotional reactivity (Greenberg and Safran, 1990; Ellis, 1999; Greenberg, 2002; Hayes et al., 2006; Mennin and Farach, 2007; Clark and Beck, 2010). Therefore it could be hypothesized that the secondary problem might negatively affect self-representation, ultimately maintaining psychopathology (Gardner et al., 1988; Hofmann, 2013).

Consistently, panic disorder is characterized by the catastrophic assessment of anxiety and its physiological correlates (i.e., anxiety sensitivity) (Clark, 1986). Similarly, social phobics often worry about the negative consequences of their anxiety in social contexts (e.g., being judged as weak or stupid; (American Psychiatric Association [APA], 2013). Despite these observations, currently there is no direct empirical evidence that the meta-emotional problem is a trans-diagnostic phenomenon, or that it plays a role in ImRs effectiveness. Indirect support of the role played by the meta-emotional problem in amplifying emotional reactivity across different pathologies comes from research on self-criticism. There is evidence that trait self-criticism is a *trans*-diagnostic phenomenon implicated in the development and maintenance of a range of psychopathologies (Schanche, 2013) as it triggers, perpetuates, and intensifies emotional reactivity (Shahar, 2013). However, the key difference between self-criticism and the meta-emotional problem is that in the latter patients only criticize themselves for having a specific emotion, whereas self-criticism refers to all aspects of a patient’s life (Couyoumdjian et al., 2016). A more direct relation between the meta-emotional problem and anxiety symptomatology has been empirically tested by (Wells, 2000) who applied his Metacognitive Therapy in the context of both generalized and social anxiety. The author found reductions in fear of negative evaluation after treatment to such anxiety disorders (Wells, 2007). Moreover, a recent study directly tested whether reducing the negative assessment of specific negative emotions related to phobic stimuli (i.e., secondary problem) also reduced the experience of the aversive emotion itself (i.e., primary problem). Results revealed that participants whose meta-emotional problem was addressed during therapy also presented a decrease in autonomic arousal (as observed by decreased heart rate and increased heart rate variability) during a second exposure to phobic stimuli (Couyoumdjian et al., 2016). Furthermore, the meta-emotional problem is considered to play a role in affective disorders. Indeed, depressive rumination, a key risk factor for clinical depression, is related to negative thinking about depressive symptoms (Nolen-Hoeksema, 1991, 2000) and fear of depressed mood and anxiety was associated with rumination and emotional avoidance (Trincas et al., 2016). Strikingly, this study revealed that the tendency to have a negative secondary reaction to distress, as measured by the *Non-Acceptance* subscale of the Difficulty in Emotion Regulation Scale (Gratz and Roemer, 2008), was strongly correlated with higher negative beliefs about emotions (i.e., that emotions are irrational). Moreover, this idea was associated with feelings of guilt, shame, embarrassment, and weakness in reaction to emotional experience (Trincas et al., 2016). In line with this finding, it has been observed that feeling ashamed and humiliated for having PTSD or guilty about intrusive thoughts in OCD are predictors of poor therapy outcomes (Gilbert and Andrews, 1998; Clohessy and Ehlers, 1999) because patients avoid seeking treatment or engaging in exposure (Leahy, 2007). Finally, it has been found that negative appraisals of the sensations, emotions, or intrusive thoughts and images that are experienced are related to depression, anxiety, PTSD, metacognitive aspects of worry, alcohol abuse, marital discord, and PD (Leahy, 2001a,b, 2002, 2003; Leahy and Kaplan, 2004). Taken together, this literature seems to support the notion that the meta-emotional problem could be a trans-diagnostic phenomenon, whereas, to the best of our knowledge, there is still a lack of direct evidence that ImRs reduces the meta-emotional problem. Intriguingly, a pilot exploration of the use of Compassion-Focused imagery (CFI) in a group of self-critical people showed a significant improvement in the reported self-soothing abilities (Gilbert and Irons, 2004). However, self-criticism has been described as a personality trait that enhances people’s frustrations and anger toward themselves in reaction to failures and setbacks (Blatt, 2004; Gilbert et al., 2004; Gilbert and Irons, 2004) while in the meta-emotional problem patients only criticize themselves for having negative emotions. Moreover, CFI is different from ImRs. Thus, our hypothesis needs further testing.

## Future Directions

Future studies could test whether: (i) ImRs as a stand-alone treatment leads to a reduction of symptoms; negative self-belief and meta-emotional problem; and (ii) the reduction of the meta-emotional problem mediates the relation between symptoms reduction and negative self-belief. Social anxiety patients would be a good target for several reasons. Firstly, the role of the meta-emotional experience is commonly acknowledged and included as part of cognitive treatments for social anxiety (DSM-5 APA; Wells, 2000, 2007). Therefore, the experimental sample receiving a session of ImRs could be compared with a control sample receiving Metacognitive Therapy (Wells, 2000). Secondly, the strength of negative self-beliefs encapsulated in aversive memories of socially anxious participants has previously been tested. A similar procedure could thus be used to extract the negative self-belief from the memory and assess its strength (Wild et al., 2007, 2008; Lee and Kwon, 2013). Several measures could be administered before and after treatments to assess the hypothesis that ImRs reduces the meta-emotional problem. Specifically, the Beliefs about Emotions Questionnaire (BAEQ; Manser et al., 2012) entails six dimensions consisting of beliefs about emotions such as: overwhelming and uncontrollable, shameful and irrational, invalid and meaningless, useless, damaging and contagious. Furthermore, the Affective Control Scale (ACS; Williams et al., 1997) was designed to assess fear of losing control over emotions or fear of behavioral reactions to emotion. The scale contains four dimensions: fear of anger, depression, anxiety, and positive emotion. However, since the meta-emotional problem refers specifically to aversive and problematic emotions, its nature and intensity can be investigated more directly by means of a semi-structured interview asking participants to define what they think about their negative emotional reaction and how much they believe such an evaluation to be true on a Likert scale (Couyoumdjian et al., 2016). Moreover, since previous results have shown that the *Non-Acceptance* subscale of the DERS was strongly correlated with higher negative beliefs about emotions, this scale could be included as a measure of emotional regulation. Finally, changes in symptoms could be assessed using measures used in previous studies such as the Social Phobia Inventory (SPIN; Connor et al., 2000), the Liebowitz Social Anxiety Scale (LSAS; Liebowitz, 1987), and the Fear of Negative Evaluation Scale (FNE; Watson and Friend, 1969). If the reduction of the meta-emotional problem plays an active role in ImRs effectiveness, a reduction of SAD symptoms; negative self-belief and meta-emotional problem should be expected in the ImRs group. This reduction should be comparable or greater than that observed in the Metacognitive Therapy group. In addition, if as we here hypothesize, negative self-belief is reduced by a reduction of the meta-emotional problem, the relation between symptoms reduction and the reduction in the strength of negative self-belief should be mediated by a reduction in the meta-emotional problem.

## Discussion of Existing Accounts

Here we proposed a meta-emotional perspective on the mechanism underlying ImRs effectiveness. Specifically, a body of literature shows ImRs to strongly impact self-representation, but how? In our view, this question should be considered when assessing the predictive power of the theories that attempted to unravel the mechanisms underlying change in ImRs.

According to Arntz and colleagues (Arntz, 2012), ImRs works by directly changing the valence associated with an unconditioned stimulus (US) corresponding to the representation of the aversive event. This theory has been termed “US-revaluation” (Davey, 1989; Arntz and Weertman, 1999), a process where fear memories are weakened by changing the meaning of such stimuli. Preliminary evidence in support of the involvement of “US-revaluation” in ImRs comes from a study showing that ImRs, added to extinction, reduced the estimated probability of the occurrence of the “US” and a reduction of the “US” negative valence (Dibbets et al., 2012). One critical point of this perspective is that saying that ImRs works by changing the valence of the memory does not necessarily imply that the meaning of the self is changed. For instance, Hagenaars and Arntz (2012) found that ImRs brought about a reduction in the development of self-blame cognitions along with a reduction in intrusion development. In our view, the latter result suggests that what is changed in the patient’s knowledge system is not just the valence of the memory but a more general system of learned goals, cognitions, and beliefs of oneself. However, it appears unclear if, according to the US-revaluation hypothesis, the reduction of negative self-blame cognition is epiphenomenal or causal with respect to the change in memory valence. It has been proposed that the expression of negative emotions could restore a more general sense of control over life, increasing self-efficacy (Arntz, 2012). However, concepts like self-esteem or self-efficacy are rather aspecific and seem neither sufficient nor necessary to explain the emergency and maintenance of psychopathology or its remission. Additionally, it has been suggested that the expression of inhibited emotions facilitates the integration of the adverse memory within the autobiographical knowledge base (Dibbets and Arntz, 2016). Again, even acknowledging the cathartic power of the expression of emotion, it seems unclear how a better integration of the adverse memory in the autobiographical knowledge base would result in the reduction of negative self-belief. Moreover, even assuming that intrusions of aversive memory, as in PTSD, are prevented by a higher integration of those memories into the autobiographical knowledge base, integration seems unnecessary in PD, where aversive memories seem perfectly integrated with strong negative beliefs towards the self (i.e., schema).

Alternatively, Brewin proposed that rather than schema change at the core of CBT there is competition for retrieval between alternative representations (Brewin, 2006). He suggested that beliefs about the self do not only take the form of abstract semantic knowledge (e.g., “*I am a failure*”) but are also underpinned by episodic memories of specific autobiographical events. Therefore, an improvement in symptoms could be expected not only by verbally reappraising negative self-belief but also preventing the retrieval of episodic memories in support of the negative semantic knowledge. Consequently, ImRs may “*draw on associative* and automatic *processes to create an alternative image in memory that shares similar sensory features but is accompanied by positive rather than negative emotions*” (Wheatley et al., 2007). Furthermore, ImRs may add new contextual information to the inflexible but more salient sensory-bound representation of the aversive event, making it more likely for the new representation to win the retrieval competition (Brewin et al., 2010). However, it is worth noting that the acquisition of new contextual information relative to external events does not appear to be strictly necessary in the traditional procedure (Arntz and Weertman, 1999).

In sum, previous accounts suggested that ImRs promotes the reduction of symptoms and of negative self-belief either by changing the valence of the aversive autobiographical memory or by reducing their accessibility. In our view, both approaches offer a plausible explanation of the observed reduction in negative self-belief, however, they are not very accurate in clarifying how the change in self-belief occurs. Additionally, none of the examined theories considered the role played by emotional appraisal in connecting the aversive memory to the negative self-belief encapsulated within. Notably, appraisals concerning emotional experiences involve individuals’ beliefs about emotions. Because working with beliefs about emotions is a fundamental part of cognitive and behavioral psychotherapies (Linehan, 1993; Wells, 2008; Clark and Beck, 2009, 2010; Leahy, 2015) we here propose a link between the change occurring in the beliefs about one’s own emotion and the change occurring in self-belief.

## Author Contributions

AM and FM substantially contributed to the conception of the work and drafting the manuscript.

## Conflict of Interest Statement

The authors declare that the research was conducted in the absence of any commercial or financial relationships that could be construed as a potential conflict of interest.
